# Supplementary material for: Compromised Cranio-Spinal Suspension in Chiari Malformation Type 1: A Potential Role as Secondary Pathophysiology
Source: J Clin Med. 2022 Dec 15;11(24):7437. doi: 10.3390/jcm11247437 (PMC9788407; doi:10.3390/jcm11247437)
Supplement: Supplementary file 1 [file jcm-11-07437-s001.zip › Video captions.pdf]

Video S1: myodural bridge complex
In this video, the myodural bridges are demonstrated pulling the dura, demonstrating their full integration into the superficial layer of dura and their role in mobilizing the dura.

Video S2: arachnoid bands
Here, the arachnoid bands are seen not only obstructing CSF flow, but also exerting direct traction on the tonsils and cervicomedullary junction which are seen functionally sus-pending these structures within the cisterna magna.

Video S3: arachnoid web tethering
An intraoperative pre-dural opening ultrasound shows a small dorsal arachnoid adhesion or web (upper left of video), which appears to tether the spinal cord to the dorsal dura. This band is seen tugging and moving the spinal cord with the impulses of systole, diastole, inspiration, and expiration, as well as direct intraoperative manipulation of the bands themselves.

Video S4: pistoning of tonsils
Ultrasound image prior to dural opening demonstrates herniated tonsils pistoning against the spinal cord, not only producing a strain on the spinal cord but tethering it to the vertebrobasilar junction, lacking any noticeable movement or CSF pulse-synchronized motion.
